# Disparities in Echinococcosis Control in Xizang, China: An Assessment Based on the Health Resource Density Index

**DOI:** 10.1155/jotm/8891867

**Published:** 2026-07-29

**Authors:** Zhiyi Wang, Quzhen Gongsang, Mingzhe Jiang, Jiaxi Lei, Pin Yang, Ying Wang, Liying Wang, Roger Frutos

**Affiliations:** ^1^ National Institute of Parasitic Diseases, Chinese Center for Tropical Diseases Research, Chinese Center for Disease Control and Prevention, Shanghai, China, chinacdc.cn; ^2^ NHC Key Laboratory of Echinococcosis Prevention and Control, Xizang Center for Disease Control and Prevention, Lhasa, China; ^3^ Intertryp, UMR17, CIRAD, Montpellier, France, cirad.fr; ^4^ Faculty of Medicine-Ramathibodi Hospital, Mahidol University, Bangkok, Thailand, mahidol.ac.th; ^5^ Faculty of Occupational Science, Universitas Airlangga, Surabaya, Indonesia, unair.ac.id; ^6^ School of Public Health, Xiamen University, Xiamen, China, xmu.edu.cn

**Keywords:** echinococcosis, health human resources, health resource density index, Xizang

## Abstract

**Background:**

Echinococcosis remains a major public health concern in Xizang, China, characterized by high endemicity in remote and underserved areas. Despite long‐term implementation of the national control program, the effectiveness of interventions may be constrained by insufficient and unevenly distributed health human resources (HHR).

**Objectives:**

To evaluate the distribution of HHR in Xizang and its association with echinococcosis prevalence at the township level using the health resource density index (HRDI).

**Methods:**

Township‐level data on echinococcosis prevalence were collected from the Xizang Center for Disease Control and Prevention. HHR data were collected from the China Health Statistics Yearbook 2022. HRDI was constructed to quantify HHR allocation by integrating population size and geographic area. Differences in HRDI across regions and production types were analyzed using the Kruskal–Wallis test with Bonferroni correction for multiple comparisons. Spatial autocorrelation analysis using Moran’s *I* and Getis‐Ord Gi∗ statistics was performed to identify clustering patterns of HRDI. Spearman’s rank correlation was used to evaluate the relationship between HRDI and echinococcosis prevalence.

**Results:**

The density of health technicians in Xizang (7.0 per 1000 population) was lower than the national average, with pronounced urban–rural disparities. Significant differences in HRDI were observed across cities (*p* < 0.05), with the lowest levels identified in Ali and Nagqu. In addition, HRDI varied significantly across production types (*p* < 0.05), with lower levels observed in semiagricultural and semipastoral areas and pastoral areas. Spatial analysis revealed significant clustering of HRDI, with low‐value clusters predominantly distributed in pastoral areas such as Ali and Nagqu. HRDI was negatively correlated with echinococcosis prevalence (rs = −0.326, *p* < 0.05).

**Conclusions:**

Insufficient and uneven distribution of HHR is associated with increased echinococcosis prevalence in Xizang. Strengthening HHR allocation in high‐endemic areas, alongside community‐based interventions and improved health service accessibility, is essential for enhancing the effectiveness of echinococcosis control programs.

## 1. Introduction

Echinococcosis is a zoonotic parasitic disease caused by the larval stages of *Echinococcus* spp. It is of widespread concern globally and has been classified by the World Health Organization as one of the neglected tropical diseases [1]. In China, the predominant forms of echinococcosis are cystic echinococcosis (CE) caused by *Echinococcus granulosus* and alveolar echinococcosis (AE) caused by *Echinococcus multilocularis*. *Echinococcus granulosus sensu lato* exhibits considerable genetic diversity in China, with multiple genotypes including G1, G3, G5, G6, G7, G8, and G10 reported, whereas *Echinococcus multilocularis* is mainly represented by the Asian and Mongolian genotypes [2, 3].

China is one of the most endemic countries for echinococcosis, bearing the largest population at risk and number of cases in the world [4, 5]. It has been estimated that the disease burden of echinococcosis in China reaches approximately 322,400 disability‐adjusted life years (DALYs), accounting for about 40% and 95% of the global burden of CE and AE, respectively [6–8]. Xizang, located in the Qinghai‐Tibet Plateau, is one of the most highly endemic regions for echinococcosis in China. A national survey conducted between 2012 and 2016 reported a prevalence of 1.66% and estimated that nearly 50,000 patients were affected in Xizang [9]. All 74 counties in Xizang are endemic for CE, among which 47 counties also have co‐endemicity of AE. At the township level, all 692 townships are endemic to echinococcosis, with the prevalence in over 80% of townships exceeding 0.1%, and 127 townships showing a prevalence above 1% [10, 11].

The transmission of *Echinococcus* spp. relies on a complex life cycle involving definitive and intermediate hosts. Eggs are excreted in the feces of definitive hosts and contaminate the environment. Intermediate hosts become infected through ingestion of these eggs, after which the larval stages develop into metacestodes within the hosts. The life cycle is completed when infected organs of domestic ungulates, such as sheep and cattle, harboring *Echinococcus granulosus* are fed to domestic dogs, or when small rodents infected with *Echinococcus multilocularis* are preyed upon by wild canids such as foxes and wolves. In China, CE is mainly maintained through a domestic transmission cycle, with domestic dogs as the primary source of infection, whereas AE is mainly maintained through a sylvatic transmission cycle, with wild canids serving as the main sources of infection. In Xizang, the large population of stray dogs and the widespread free‐ranging practices of domestic dogs provide favorable ecological conditions for the transmission of both CE and AE. The transmission of echinococcosis is a complex process influenced by multiple factors, which can be broadly categorized into environmental, biological, and socioeconomic determinants [1, 12–16]. Environmental factors, including geographical and climatic conditions, affect the survival of parasite eggs and the spatial distribution of hosts [1, 13]. Biological factors, particularly the density and infection status of definitive hosts such as dogs and foxes, as well as intermediate hosts such as livestock and small rodents, play a crucial role in maintaining the transmission cycle [14, 15]. In addition, socioeconomic factors, including cultural practices, economic conditions, levels of health knowledge, and patterns of human production and daily activities, also contribute to the transmission and persistence of echinococcosis [16].

To prevent and control echinococcosis, the central government transfer payment project was initiated nationwide in 2005, with all counties in Xizang being gradually incorporated into this project from 2008 [11]. Despite considerable financial investment from the government, echinococcosis control in remote areas remains a persistent challenge [17]. The effective implementation of key control measures—such as dog registration and management, deworming of domestic and wild canids, prohibition of feeding infected livestock offal to dogs, and health education—primarily relies on the efforts of grassroots health workers, particularly health technicians, rural doctors, and township veterinarians.

However, a severe imbalance in the health human resources (HHR) in Xizang significantly hinders these prevention and control efforts. Compounded by the region’s vast territory and sparse population, the limited HHR is predominantly concentrated in urban and peri‐urban areas, leading to critically inadequate health services in many remote agricultural and pastoral communities [18]. This imbalance in HHR reflects a common challenge across China, particularly manifesting as an urban–rural disparity, and is closely linked to disparities in health service utilization and accessibility [19–22].

The health resource density index (HRDI) is a comprehensive measure for evaluating the distribution of regional health resources, integrating both the service radius coverage and population size [23, 24]. This metric is particularly suitable for application in Xizang. This study analyzed the relationship between the prevalence of echinococcosis and the HRDI, providing a basis for optimizing the allocation of HHR and improving prevention and control measures. It is recommended to adjust the allocation of HHR in Xizang and enhance prevention and control strategies to further curb the spread of echinococcosis.

## 2. Materials and Methods

### 2.1. Data Sources

Data on health workers in Xizang, as well as in three provinces at the same latitude (Sichuan, Hubei, and Jiangsu) representing western, central, and eastern China, along with national‐level aggregates, were extracted from the China Health Statistical Yearbook 2022 [25], showing little change in recent years.

Medical and health institutions include all hospitals, primary healthcare institutions, specialized public health institutions, and other healthcare facilities. Health workers comprise all employees working within these institutions, categorized as health technicians, village doctors, village health aides, other technical staff, administrative staff, and support staff. Health technicians include practicing physicians, practicing assistant physicians, registered nurses, pharmacists (including assistants), laboratory technicians (including assistants), medical imaging technicians (including assistants), public health inspectors, and trainees. Village doctors are defined as personnel providing services in clinics holding doctor practice certification, while village health aides perform comparable duties without certification.

Demographic data for 692 townships of 74 endemic counties in Xizang were obtained from the population survey released by the National Bureau of Statistics. Clinically diagnosed and confirmed cases of human echinococcosis (including CE and AE) were collected from the annual report system of the Xizang Center for Disease Control and Prevention. Human echinococcosis cases were diagnosed by B‐ultrasonography according to China’s official “Diagnostic criteria for echinococcosis” (WS 257–2006), which is in line with that of the World Health Organization. The prevalence of human echinococcosis, representing confirmed cases (including both incident and prevalent cases) based on the population survey of individuals aged ≥ 2 years across these 692 townships, reflects both the disease burden and the transmission risk. The number of health workers involved in the prevention and control activities of echinococcosis in each township was collected by structured questionnaires, covering health technicians, village doctors, village health aides, and other relevant personnel. Townships were classified as urban areas, agricultural areas, semiagricultural and semipastoral areas, and pastoral areas based on production types.

### 2.2. Statistical Analysis

Using the coordinate transformation function in ArcGIS 10.8 (Esri Inc., Redlands, CA, USA), the map data of Xizang were converted to the UTM coordinate system, and the area of each township was calculated. Data on various health workers involved in the prevention and control of echinococcosis in each township were collected by structured questionnaires and then integrated. The HRDI has been widely used to evaluate the equity of health resource allocation in previous studies [23, 24]. The HRDI for each of the 692 townships in Xizang was calculated using the following formula:

(1) HRDI=is×ip.

Here, *i* represents the number of health workers in the township, *s* represents the area of the township, and *p* represents the permanent resident population. The normality of HRDI distribution was assessed using the Kolmogorov–Smirnov (K–S) test. Differences in HRDI across cities and production types were analyzed with the Kruskal–Wallis (K–W) test, followed by Bonferroni correction. The association between HRDI and human echinococcosis prevalence was evaluated using Spearman’s rank correlation. All statistical analyses were performed using SPSS, version 20.0 (IBM Corp., Armonk, NY, USA).

Spatial autocorrelation of township‐level HRDI in Xizang was analyzed using ArcGIS 10.8 (Esri Inc., Redlands, CA, USA). Global spatial autocorrelation was assessed using Moran’s *I* index, which ranges from −1 to 1. A positive index indicates positive spatial autocorrelation (clustering of similar values), with magnitude reflecting the strength of clustering. A negative index suggests negative spatial autocorrelation (dispersion or dissimilarity between neighboring areas), with lower values indicating greater spatial heterogeneity. An index close to zero denotes a spatially random pattern. Local spatial autocorrelation was examined using hot spot analysis (Getis‐Ord Gi∗) to identify statistically significant spatial clusters of high values (hot spots) and low values (cold spots). These methods are widely used in spatial epidemiology to detect spatial clustering patterns [26, 27].

Statistical significance was defined as *p* < 0.05 for all analyses.

### 2.3. Ethical Statement

This survey was approved by the Ethical Review Committee of the National Institute of Parasitic Diseases, Chinese Center for Disease Control and Prevention (No. 20160810). All participants were informed of the content and purpose of the investigation and examination, potential complications, consequences, as well as benefits before examination. Those who agreed to participate were required to sign written informed consent forms. All participants were given feedback. All echinococcosis diagnosed patients provided written agreements to participate and were provided with free drug treatment or subsidized surgical costs.

## 3. Results

### 3.1. HHR in Xizang

In 2022, a total of 1655 medical and health institutions were recorded in Xizang, employing 42,311 health workers, far below the national average (451,141). Among these, 18,509 were urban health workers, and 23,802 were rural health workers, compared with the national averages of 257,232 in urban areas and 193,586 in rural areas. Of the total health workers, 25,607 were health technicians, accounting for 60.52% of all health workers; 2321 were other technical staff (5.48%); 1003 were administrative staff (engaged exclusively in management) (2.37%); 2923 were support staff (6.91%); and 10,457 were village doctors and village health aides (24.71%).

**FIGURE 1:**
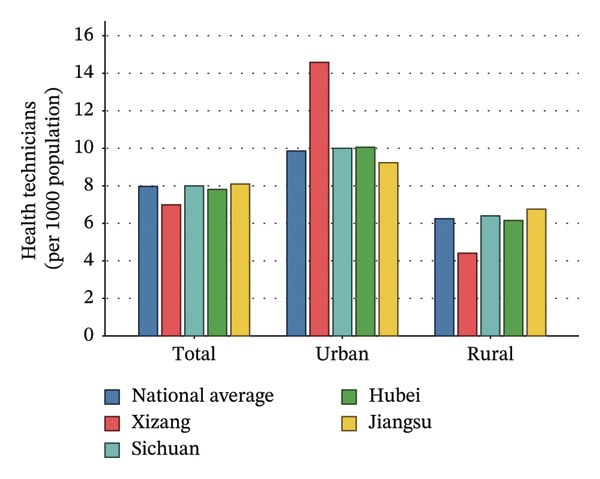
Number of health technicians per 1000 population in Xizang, selected provinces, and the national average.

In Xizang, the density of health technicians was 7.0 per 1000 population, below both the national average (7.97) and the rates in provinces at the similar latitude: Sichuan (8.04), Hubei (7.83), and Jiangsu (8.13). Despite the rural population (2.75 million) exceeding the urban population (0.91 million), health technicians were disproportionately concentrated in urban areas (14.62 per 1000 population) compared to rural areas (4.46 per 1000) (Figure 1).

The density of practicing physicians (including assistants) in urban areas (6.14 per 1000) was 3.36 times that in rural areas (1.83 per 1000), representing a 235.5% higher rate. Similarly, the registered nurse density in urban areas (5.43 per 1000) was 5.27 times the rural rate (1.03 per 1000), or 427.2% higher (Table 1).

**TABLE 1:** Number of practicing physicians and registered nurses (per 1000 population) in different regions.

	Xizang	Sichuan	Hubei	Jiangsu	National average
Practicing physicians (including assistants)					
Urban areas	6.14	3.72	3.67	3.52	3.73
Rural areas	1.83	2.39	2.35	2.82	2.42
Total	2.90	2.99	2.91	3.21	3.04
Registered nurses					
Urban areas	5.43	4.75	4.94	4.24	4.58
Rural areas	1.03	2.77	2.76	2.88	2.64
Total	2.13	3.66	3.68	3.63	3.56

Village clinics serve as the primary healthcare providers in rural areas. In terms of total number of health workers in village clinics, Xizang had a rate of 4.51 per 1000 population, which was more than twice the national average of 1.83 and more than twice the rates of the three provinces at the same latitude (Sichuan 2.02, Hubei 1.94, Jiangsu 1.92). However, the number of practicing physicians (including assistants) in Xizang village clinics was 0.46 per 1000 population, and the number of registered nurses was 0.24 per 1000, both of which were relatively low. At the same time, the number of village doctors and health aides in Xizang reached 3.81 per 1000 population, which was almost 4 times the national average of 0.94 (Table 2).

**TABLE 2:** Number of various health workers (per 1000 population) in village clinics in different regions.

Category	Xizang	Sichuan	Hubei	Jiangsu	National average
Practicing physicians (incl. assistants)	0.46	0.61	0.63	0.96	0.64
Registered nurses	0.24	0.23	0.43	0.40	0.26
Village doctors and health aides	3.81	1.18	0.89	0.56	0.94
Total	4.51	2.02	1.94	1.92	1.83

### 3.2. HRDI in Xizang

To characterize the overall distribution of HRDI, a single‐sample K–S test was performed, indicating a non‐normal distribution (*p* < 0.05). Therefore, the median (P25, P75) was used to describe the distribution of HRDI. Differences in HRDI across the seven cities were assessed using the K–W test, followed by pairwise comparisons with Bonferroni correction (Table 3). Pairwise comparisons showed that HRDI in Ali and Nagqu was significantly lower than that in most other cities (*p* < 0.05). In contrast, HRDI values in Lhasa and Shannan were relatively higher, with no statistically significant difference observed between them (*p* = 0.289). No significant differences were found between Linzhi and Shigatse, or between Linzhi and Changdu (*p* > 0.05). Overall, these findings indicate that Ali and Nagqu experienced the most pronounced shortages of health resources among the seven cities.

**TABLE 3:** Pairwise comparisons of township‐level HRDI across seven cities in Xizang.

City	Median (P25–P75)	Lhasa	Shigatse	Changdu	Linzhi	Shannan	Nagqu	Ali
Lhasa	0.0077 (0.0044–0.0133)	1.000	0.007	< 0.001	< 0.001	0.289	0.002	< 0.001
Shigatse	0.0050 (0.0034–0.0076)	0.007	1.000	< 0.001	0.055	0.110	< 0.001	< 0.001
Changdu	0.0034 (0.0025–0.0046)	< 0.001	< 0.001	1.000	0.060	< 0.001	< 0.001	< 0.001
Linzhi	0.0041 (0.0030–0.0062)	< 0.001	0.055	0.060	1.000	0.004	< 0.001	< 0.001
Shannan	0.0054 (0.0043–0.0089)	0.289	0.110	< 0.001	0.004	1.000	< 0.001	< 0.001
Nagqu	0.0026 (0.0018–0.0037)	0.002	< 0.001	< 0.001	< 0.001	< 0.001	1.000	0.148
Ali	0.0021 (0.0010–0.0029)	< 0.001	< 0.001	< 0.001	< 0.001	< 0.001	0.148	1.000

Townships in Xizang can be divided into urban areas, agricultural areas, semiagricultural and semipastoral areas, and pastoral areas according to production type. To examine the association between production type and HRDI, the K–W test was performed, followed by pairwise comparisons with Bonferroni correction (Table 4). The results showed significant differences in HRDI across different production types (*p* < 0.05). Pairwise comparisons indicated that HRDI in semiagricultural and semipastoral areas and pastoral areas was significantly lower than that in agricultural areas (*p* < 0.05). HRDI in pastoral areas was also significantly lower than that in urban areas (*p* < 0.05). However, no statistically significant difference was observed between urban areas and semiagricultural and semipastoral areas (*p* > 0.05). Overall, these findings suggest that townships in semiagricultural and semipastoral areas and pastoral areas experienced relatively lower levels of health resource allocation.

**TABLE 4:** Pairwise comparisons of township‐level HRDI by production type in Xizang.

Production type	Median (P25–P75)	Urban areas	Agricultural areas	Semiagricultural and semipastoral areas	Pastoral areas
Urban areas	0.0069 (0.0039–0.0686)	1.000	0.593	0.100	< 0.001
Agricultural areas	0.0061 (0.0046–0.0095)	0.593	1.000	< 0.001	< 0.001
Semiagricultural and semipastoral areas	0.0044 (0.0031–0.0069)	0.100	< 0.001	1.000	< 0.001
Pastoral areas	0.0026 (0.0017–0.0039)	< 0.001	< 0.001	< 0.001	1.000

According to the distribution characteristics, the HRDI of townships was divided into four levels: HRDI < 0.0025, 0.0025 ≤ HRDI < 0.0050, 0.0050 ≤ HRDI < 0.0075, and 0.0075 ≤ HRDI. Lower HRDI levels indicated more scarce HHR. The distribution of HRDI in townships of each city is shown in Table 5.

**TABLE 5:** Distribution of townships by HRDI level across cities in Xizang.

City	Level 1		Level 2		Level 3		Level 4		Total
	*N*	Percentage (%)	*N*	Percentage (%)	*N*	Percentage (%)	*N*	Percentage (%)	
Lhasa	6	9.2	14	21.5	11	16.9	34	52.3	65
Shigatse	20	9.9	80	39.4	50	24.6	53	26.1	203
Changdu	34	24.6	72	52.2	18	13.0	14	10.1	138
Linzhi	9	17.0	24	45.3	13	24.5	7	13.2	53
Shannan	6	7.3	29	35.4	22	26.8	25	30.5	82
Nagqu	53	46.5	49	43.0	6	5.3	6	5.3	114
Ali Region	24	64.9	9	24.3	3	8.1	1	2.7	37
Total	152	22.0	277	40.0	123	17.8	140	20.2	692

**FIGURE 2:**
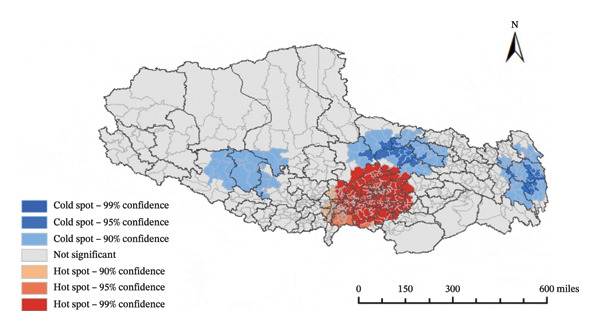
Spatial distribution of HRDI hot spots and cold spots at the township level in Xizang.

Spatial analysis of township‐level HRDI revealed significant geographic clustering patterns. Global Moran’s I demonstrated strong positive spatial autocorrelation (Moran’s *I* = 0.445, *Z* = 52.35, *p* < 0.05), confirming nonrandom aggregation of HRDI values across townships. Local Getis‐Ord Gi hot spot analysis further identified statistically significant clusters, with high‐HRDI hot spots concentrated predominantly in south‐central Xizang and low‐HRDI cold spots prevalent in eastern, central, and western townships. These results indicate spatially uneven health resource allocation, with intensive aggregation in the south‐central core region (Figure 2).

### 3.3. Correlation Analysis Between HRDI and Prevalence of Echinococcosis in Xizang

The distribution of echinococcosis prevalence across the 692 townships in Xizang deviated from normality (*p* < 0.05). Therefore, Spearman’s rank correlation analysis was performed to examine the association between HRDI and echinococcosis prevalence. A significant negative correlation was observed (rs = −0.326, *p* < 0.05), indicating that areas with lower health resource density tended to have higher echinococcosis prevalence.

**FIGURE 3:**
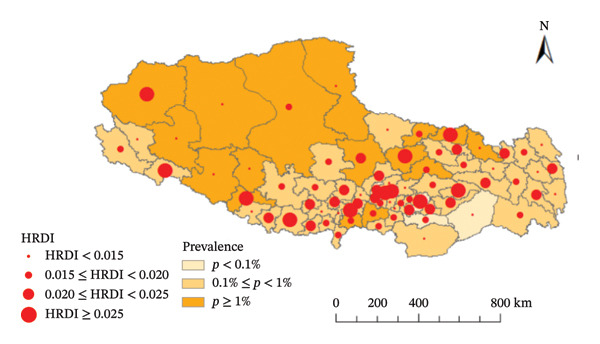
Spatial distribution of HRDI and echinococcosis prevalence at the county level in Xizang.

At the county level, HRDI demonstrated a normal distribution with a mean of 0.0181 ± 0.0077. ArcGIS 10.8 was used to visualize the echinococcosis prevalence and HRDI in each county. Echinococcosis prevalence and HRDI maps in each county of Xizang were obtained, as shown in (Figure 3). Counties with higher echinococcosis prevalence exhibited lower HRDI, reinforcing the significant negative association between health workforce density and echinococcosis prevalence.

## 4. Discussion

The Xizang Autonomous Region, abbreviated as “Xizang,” is located on the southwest border of China and the southwest part of the Qinghai‐Tibet Plateau. It spans about 1.20 million square kilometers, accounting for about one‐eighth of China’s total area [28].

This study compared the average HHR in Xizang with three provinces from the western, central, and eastern regions of China at the same latitude, as well as with the national average. In 2022, Xizang had a permanent population of 3.66 million, with 7.00 health technicians, 2.90 practicing physicians (including assistants), and 2.13 registered nurses per 1000 people [29]. Notably, the density of health technicians (7.0 per 1000 population) was below the national average (7.97), while its practicing physicians (including assistants) density (2.90 per 1000) was slightly lower than the national average (3.04), though comparable to the average level in western provinces (2.88) [25]. Xizang’s overall HHR remains lower than that of the other provinces. In addition, the distribution of health technicians is imbalanced between urban and rural areas. The majority of HHR are concentrated in urban areas, leading to a shortage of HHR in some rural regions. Such regional disparities are a major source of inequity in public health resource allocation efficiency in rural China [30]. Among the health workers serving rural population in Xizang, the number of practicing physicians (including assistants), registered nurses, and rural registered general practitioners is lower. In contrast, the number of health technicians, village doctors, and health aides in village clinics is much higher than that in the three comparison provinces and the national average. Regarding personnel quality, practicing physicians possess higher qualifications than staff in village clinics, which affects the quality of rural health service [31]. Therefore, this imbalance in both quantity and quality also limits the level of healthcare available to the rural population. Beyond urban–rural disparities in HHR quantity and quality, inter‐regional inequality in health worker distribution also exists in Xizang, aligning with previous studies [32, 33]. Given Xizang’s vast territory and low population density, this study calculated a comprehensive index of HHR, the HRDI, based on geographical and population data to assess allocation. The distribution of HRDI at the township level is imbalanced, with higher values concentrated in central areas, while lower‐value areas require more attention and resource allocation. Significant differences in HRDI exist among different municipal cities, with Ali and Nagqu having lower values than other cities. Therefore, HHR allocation should be prioritized toward Ali and Nagqu. The persistent urban–rural disparity in HHR highlighted by the study findings warrants in‐depth investigation. This disparity is primarily attributable to Xizang’s unique high‐altitude environment, characterized by high elevation, extreme climatic conditions (e.g., low temperatures, hypoxia, intense radiation), inaccessible terrain, relative economic underdevelopment, and underdeveloped educational infrastructure. These factors collectively create significant challenges in attracting and retaining qualified health technical personnel, particularly those trained in urban centers. Simultaneously, local capacity and resources for training health workers are severely limited, making it difficult to adequately meet demand. Furthermore, insufficient economic incentives, a scarcity of professional development opportunities, and deficient basic living and working infrastructure (e.g., housing, transportation, communications, medical equipment) at the grassroots level exacerbate the problem, driving the concentration of HHR in urban areas and perpetuating the shortage in rural primary care.

Xizang is an endemic area for echinococcosis. Based on data from township‐level administrative divisions, this study analyzed the correlation between HRDI and the prevalence of echinococcosis in Xizang. Echinococcosis imposes a significant burden on patients, and implementing intervention measures can reduce prevalence and generate considerable economic benefits [34]. The Chinese government has initiated a national program for the prevention and control of echinococcosis and has invested substantial resources in Xizang [35]. Furthermore, a three‐tier leadership structure (province, city, and county levels) has been established for echinococcosis control, ensuring unified and efficient coordination [36]. Effective utilization of resources and implementation of echinococcosis prevention and control measures require sufficient HHR. Our analysis shows a negative correlation between HRDI and echinococcosis prevalence, indicating that areas with higher disease burden tend to have fewer HHR. In counties with low HRDI levels, echinococcosis control is compromised. Key prevention and control measures for echinococcosis, including population screening, dog management, wild canine deworming, and livestock slaughter oversight, rely heavily on health workers and veterinarians from township health centers and village clinics, especially for dog management, livestock slaughter management, livestock vaccination, and rodent control. Additionally, health education and medical record management also require the involvement of these primary health workers [37]. However, the vastness of rural areas and the shortage of HHR limit the efficiency of prevention and control efforts for echinococcosis. Therefore, regions with high prevalence need to increase investment in HHR, especially in rural areas.

The persistent shortage of HHR in Xizang presents significant challenges that are unlikely to be alleviated in the near future. This reality necessitates the implementation of community‐driven control strategies to complement conventional medical interventions, particularly through health education initiatives to mobilize local residents in prevention efforts. Crucially, several high‐impact interventions—including praziquantel administration for dogs, proper disposal of dewormed canine feces [9], and visual identification and safe handling of infected viscera during household livestock slaughter—can be mastered and directly implemented by trained local residents through systematic training. The implementation of echinococcosis control measures in Xizang cannot rely solely on specialized health personnel executing all activities; instead, the core strategy should involve training residents to recognize the critical importance of developing hygienic habits (e.g., handwashing, avoiding untreated water) and standardized practices (e.g., safe disposal of infected viscera), thereby integrating them into the prevention workforce as frontline executors of these key measures. This approach substantially reduces reliance on specialized HHR. Successful implementation depends on two fundamental prerequisites: enhancing public awareness of echinococcosis transmission risks and promoting active community engagement through culturally appropriate behavioral interventions. Notably, in regions with prevalent household livestock slaughter, training residents to identify and properly dispose of infected organs may represent a more feasible approach than large‐scale monitoring. When coordinated by health workers through local networks (e.g., village leaders, schools), these community‐based measures could establish a sustainable prevention framework. Therefore, while continued investment in HHR remains essential, empowering communities through knowledge dissemination and skills training represents a cost‐effective and practical strategy for echinococcosis control. This dual approach—strengthening both professional healthcare capacity and community engagement—offers a pragmatic solution to current systemic constraints in disease prevention.

## 5. Conclusions

Based on our findings, Xizang must increase its allocation of HHR. Distribution should consider not only population size but also service radius coverage. Optimizing urban–rural distribution equity will significantly improve health service accessibility. For sustainable echinococcosis control, effective implementation hinges on mobilizing grassroots personnel—including village doctors, veterinarians, and community volunteers—to deliver targeted health education while providing practical training in core interventions. Some key measures such as dog registration and management, regular praziquantel treatment, and proper disposal of dog feces after deworming are essential for reducing environmental exposure to echinococcus eggs. Ultimately, adopting a strategy of social mobilization and community‐based prevention and control is the most effective way to tailor echinococcosis prevention and treatment to local conditions.

## Author Contributions

Zhiyi Wang: conceptualization (equal); data curation (equal); formal analysis (equal); investigation (equal); project administration (lead); supervision (lead); writing–original draft (lead). Quzhen Gongsang: conceptualization (equal); formal analysis (equal); supervision (equal). Mingzhe Jiang: formal analysis (equal); writing–original draft (equal). Jiaxi Lei: formal analysis (equal). Pin Yang: formal analysis (equal). Ying Wang: formal analysis (equal). Liying Wang: conceptualization (equal); data curation (lead); formal analysis (equal); investigation (equal); project administration (equal); writing–review and editing (equal). Roger Frutos: formal analysis (equal); writing–review and editing (equal).

## Funding

This study was funded by NHC Key Laboratory of Echinococcosis Prevention and Control (no. 2024WZK1001).

## Ethics Statement

This survey was approved by the Ethical Review Committee of the National Institute of Parasitic Diseases, Chinese Center for Disease Control and Prevention (No. 20160810). The performed activities were all within the scope of the national project for echinococcosis control. All participants were informed of the content and purpose of the investigation and examination, potential complications, and consequences, as well as benefits before examination. Those who agreed to participate were required to sign written informed consent forms. All participants were given feedback. All patients diagnosed with echinococcosis provided written agreements to participate and were provided with free drug treatment or subsidized surgical costs.

## Conflicts of Interest

The authors declare no conflicts of interest.

## Data Availability

The datasets generated and analyzed during the current study are not publicly available due to participant confidentiality and privacy concerns but are available from the corresponding author upon reasonable request (email: wangliyingcdc@163.com).
